# Nasopharyngeal Leiomyosarcoma: Diagnostic Challenges and Management of a Rare Head and Neck Sarcoma

**DOI:** 10.7759/cureus.116104

**Published:** 2026-09-11

**Authors:** Timothy Morley, Sarah Ridge, Jared Bunevich, Nicole M Fowler, Dwight E Heron

**Affiliations:** 1 Department of Otolaryngology - Head and Neck Surgery, Bon Secours Mercy Health, Youngstown, USA; 2 Department of Otolaryngology - Head and Neck Surgery, University Hospitals Cleveland Medical Center, Cleveland, USA; 3 Department of Radiation Oncology, Bon Secours Mercy Health, Youngstown, USA

**Keywords:** head and neck oncology, leiomyosarcoma, nasopharyngeal leiomyosarcoma, otolaryngology-head and neck surgery, sarcoma

## Abstract

Leiomyosarcoma (LMS) is a rare, smooth-muscle malignancy and an uncommon sinonasal tumor, often diagnosed at a late stage due to non-specific symptoms and complex anatomy. We report an 82-year-old man with a two-month history of facial pain, epistaxis, and right-sided hearing loss. Imaging and endoscopic evaluation showed a friable mass involving the posterior nasal cavity and nasopharynx. Biopsy confirmed a giant cell-rich spindle cell LMS. He underwent endoscopic resection with positive margins. Postoperative complications and comorbidities delayed the initiation of adjuvant radiation therapy. Rapid local recurrence soon rendered the tumor unresectable, with extension into the hard palate and oropharynx. Clinical decline precluded further therapeutic interventions, and the patient died six months after the initial onset of symptoms. This case report documents the aggressive nature of primary nasopharyngeal LMS and its propensity for early recurrence. Complete resection with negative margins is critical but often limited by anatomy. Adjuvant radiation therapy may improve local control in margin-positive disease, although the evidence is largely retrospective. Early recognition and timely multidisciplinary treatment are essential in the management of this disease.

## Introduction

Sarcomas are a heterogeneous group of malignant neoplasms of mesenchymal origin, arising in bone and soft tissues. They comprise approximately 1% of adult malignancies and 3% of pediatric cancers. Head and neck sarcomas are particularly uncommon, representing roughly 3% of all malignancies in this anatomic region and account for 4% to 10% of all sarcomas in adults [1]. Due to their rarity and non-specific presentation, these tumors are frequently misdiagnosed. Leiomyosarcoma (LMS) is a malignant tumor derived from smooth muscle cells, constituting approximately 7% of all soft tissue sarcomas. It most commonly arises in the gastrointestinal tract, uterus, and retroperitoneum. Primary LMS of the sinonasal tract (SNT) is exceedingly rare, with an estimated incidence of 1.5% to 2.3% among sinonasal neoplasms. Within the SNT, the nasal cavity and paranasal sinuses are the most frequently affected sites [2]. In this report, we present a rare case of primary LMS of the nasopharynx in an 82-year-old male, accompanied by a comprehensive review of the existing literature on LMS arising in the sinonasal region.

## Case presentation

An 82-year-old male presented with a two-month history of progressive facial pain and pressure, frequent recurrent epistaxis, and decreased right auditory acuity. He had previously completed two courses of empiric antibiotics prescribed by his primary care physician without meaningful resolution of symptoms. Due to his persistent complaints, a computed tomography (CT) (Figure 1A) and a contrast-enhanced magnetic resonance imaging (MRI) (Figures 1B-1D) scan of the sinuses were obtained, which demonstrated a right-sided nasal cavity mass with inferior extension into the nasopharynx.

**Figure 1:**
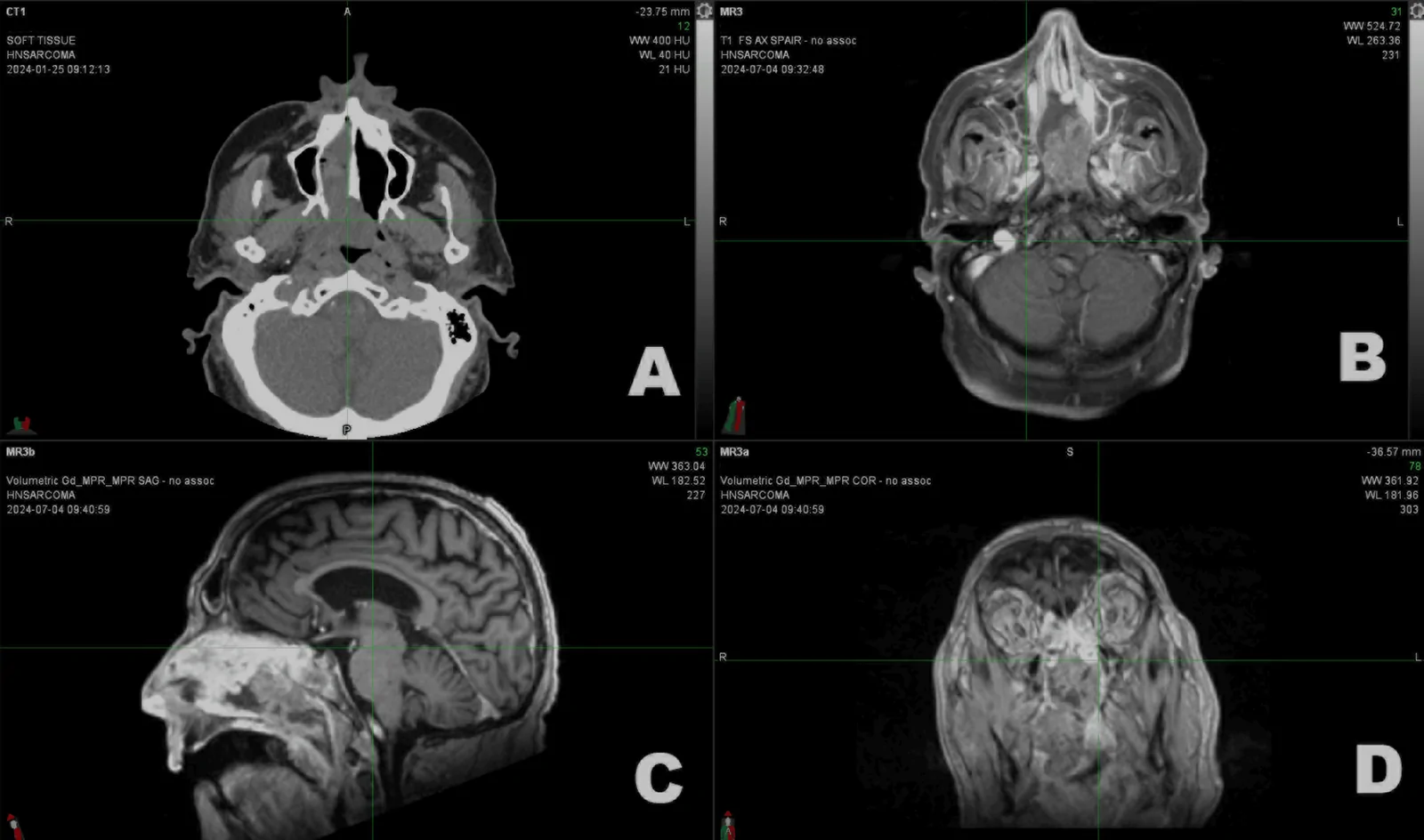
Diagnostic imaging of the head and neck (A) Axial computed tomography (CT); (B-D) axial, sagittal, and coronal magnetic resonance imaging (MRI).

He was subsequently referred to otolaryngology for further evaluations and management. In-office flexible fiberoptic nasopharyngolaryngoscopy revealed a well-demarcated, erythematous 2.4 x 1.6 x 3.4 cm nasal mass located along the right nasal floor between the nasal septum and inferior turbinate, though only the inferior aspect of the tumor could be visualized by this route. Evidence of recent tumor-associated bleeding was observed. The patient was subsequently taken to the operating room for a diagnostic nasal endoscopy, which identified a friable, exophytic lesion originating from the posterior edge of the right posterior soft palate and extending to involve the posterior nasal septum. The mass extended into the nasopharynx, occupying the right Fossa of Rosenmuller and partially obstructing the left nasal cavity. There was no radiographic evidence of paranasal sinus involvement bilaterally, and the mucosa overlying the inferior and middle turbinates appeared intact. Biopsy revealed a giant cell-rich spindle cell sarcoma consistent with LMS.

The patient was discussed at a multidisciplinary tumor board and subsequently underwent endoscopic resection via an intranasal approach, including a partial palatectomy and partial posterior septectomy, performed under image guidance. Final pathology confirmed a giant cell-rich spindle cell sarcoma consistent with an LMS. The tumor involved the nasopharynx, right and left hard palate, and left soft palate. Notably, soft tissue margins were positive on the left soft palate specimen.

Postoperative recovery was complicated by acute oronasal bleeding requiring operative intervention for hemostasis. During preparation for intubation in the operating room, the patient experienced an episode of acute oxygen desaturation. A rapid sequence intubation was attempted, but visualization was compromised due to blood clots. A blind intubation attempt resulted in esophageal intubation. Following removal of the endotracheal tube and resumption of bag-mask ventilation, the patient experienced pulselessness. Cardiopulmonary resuscitation was initiated, and return of spontaneous circulation was achieved within two to three minutes. The patient was subsequently intubated successfully, and the procedure for hemostasis control was completed without further complications. He was then referred to Radiation Oncology for postoperative management.

The initiation of post-operative radiation therapy was delayed due to a complex post-operative course including catheter ablation for persistent atrioventricular nodal reentrant tachycardia, dental evaluation for obturator placement, and patient-driven delays related to second opinion consultations. He was ultimately evaluated by Radiation Oncology approximately seven weeks postoperatively. In-office flexible fiberoptic laryngoscopy at the time demonstrated post-surgical changes with granulomatous tissue emanating from the right inferior turbinate and nasopharynx. The bilateral eustachian tubes appeared to be patent and without evidence of distortion of the Fossa of Rosenmuller. No gross recurrence was appreciated. Laryngoscopy further revealed normal-appearing piriform sinuses, surrounding laryngeal structures, and the base of tongue. Both vocal cords were fully mobile.

However, at a follow-up visit with the otolaryngology head and neck surgery team approximately 10.5 weeks postoperatively, prior to the initiation of adjuvant radiation therapy, recurrent disease was identified. In-office nasolaryngoscopy revealed a vascularized mass emanating from the bilateral soft palate which obstructed visualization beyond the middle meatuses, and a CT scan of the soft tissue neck demonstrated a 3.0 x 4.0 x 3.0 cm nasopharyngeal/oropharyngeal mass with interval development of erosion into the posterior right hard palate protruding into the oral cavity (Figures 2A-2B). Given the extent of local recurrence and anatomic involvement, the tumor was deemed unresectable by the surgical team.

**Figure 2:**
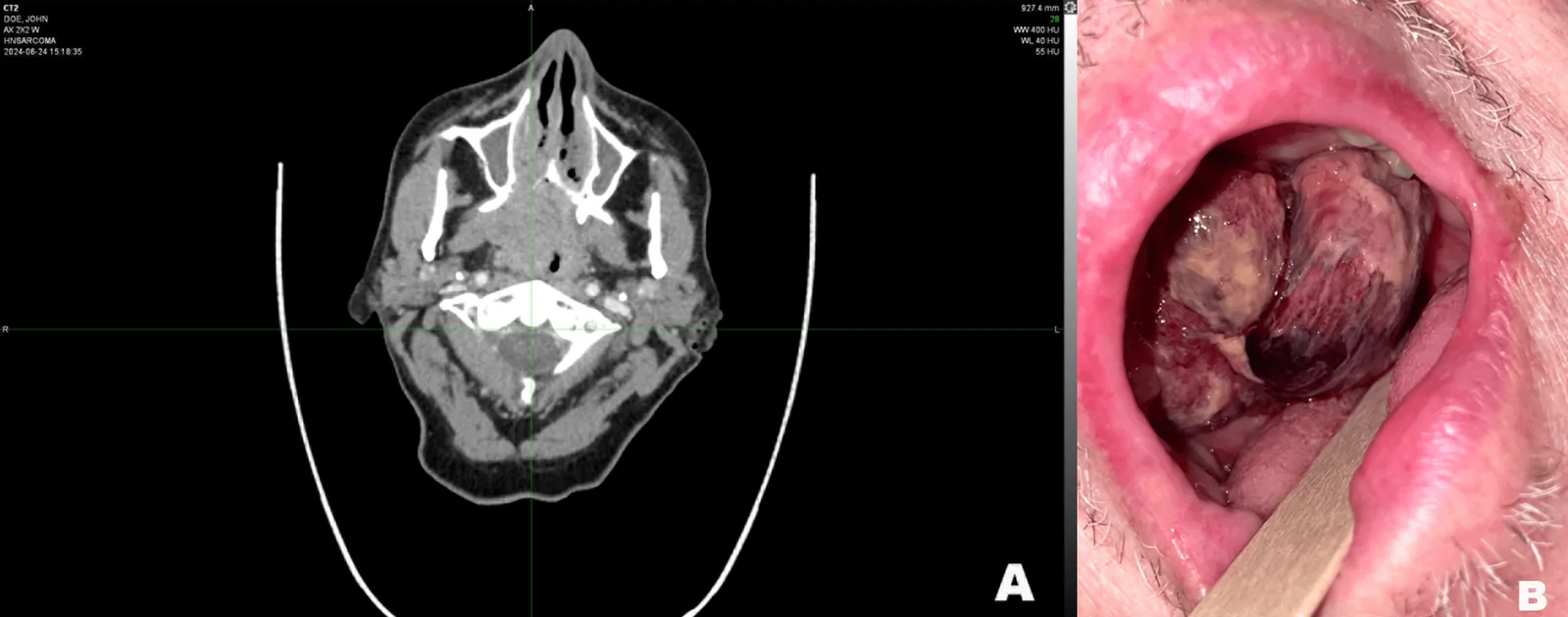
Diagnostic axial CT (A) and intraoral examination demonstrating recurrent tumor (B)

The patient was re-evaluated by the radiation oncology team to discuss potential palliative treatment options. However, plans for radiation therapy were discontinued shortly thereafter due to recurrent episodes of oxygen desaturation in the supine position, presumed to result from tumor-related oropharyngeal airway obstruction. Considering his progressive clinical decline and poor functional status, the decision was made to pursue hospice care. The patient passed away several days later under comfort-focused management, approximately six months after the onset of symptoms and three months after diagnosis.

## Discussion

This case illustrates the aggressive clinical course of primary nasopharyngeal leiomyosarcoma (NPLS), a rare subtype of soft tissue sarcoma of the head and neck region. While head and neck sarcomas account for approximately 3% of malignancies in this anatomic region and 4% to 10% of all adult sarcomas, primary NPLS represents a particularly uncommon entity within this group. Established risk factors for sarcomagenesis in the head and neck include occupational exposure to leather and textile dust, formaldehyde, chlorophenols, and cutting oils [3].

The clinicopathological and molecular features of NPLS remain incompletely defined, largely due to its rarity and histologic overlap with other spindle cell neoplasms. It is hypothesized that aberrant differentiation of the smooth muscle precursors within the primitive mesenchyme may underlie the pathogenesis of these tumors [4]. Histological diagnosis requires careful evaluation, as morphologic features vary with degree of differentiation. However, a minimum diagnostic criterion includes the presence of intersecting fascicles of elongated, brightly eosinophilic spindle cells with vesicular, ovoid- to cigar-shaped nuclei. Immunohistochemical staining typically reveals strong positivity for smooth muscle markers such as desmin and/or smooth muscle actin (SMA) (Figures 3A-3B), which support a diagnosis of LMS [5].

**Figure 3:**
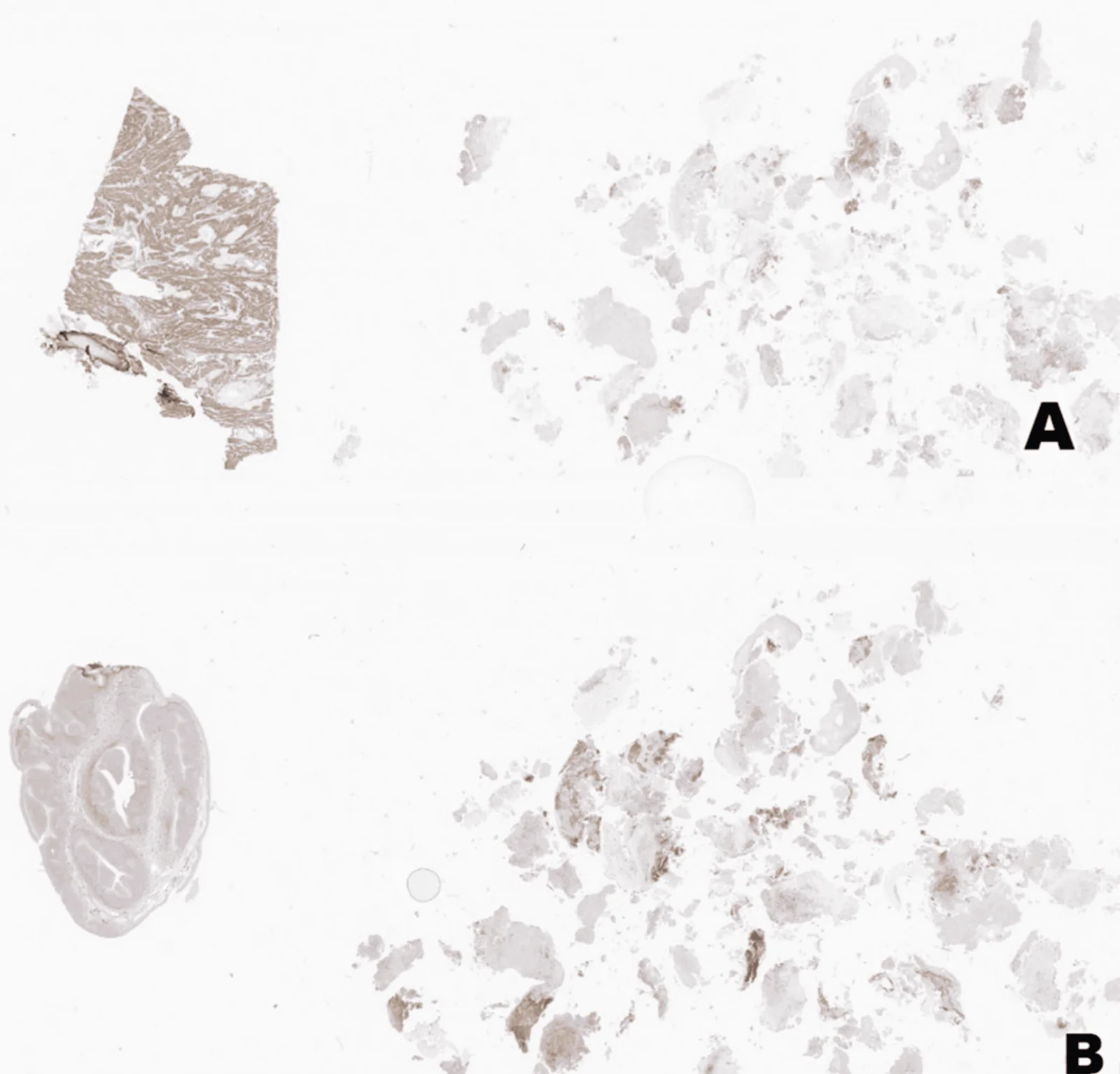
Immunohistochemical staining for desmin (A) and smooth muscle actin (SMA) (B)

Surgical resection remains the cornerstone of LMS of the SNT. Due to the extreme rarity of NPLS, treatment guidelines are not based on randomized trials but rather on case reports, single-institution series, and analyses of large databases such as the National Cancer Database (NCDB). Reported five-year survival rates for sinonasal sarcomas, including NPLS, range from 61% to 100%, with outcomes dependent on tumor site, histologic grade, extent of disease, margin status, and the use of adjuvant therapies [6,7].

Because primary NPLS is exceptionally rare, management is best individualized through multidisciplinary tumor board review. Consensus among head and neck surgeons, radiation oncologists, medical oncologists, neuroradiologists, and sarcoma-experienced pathologists is essential to confirm the diagnosis, assess resectability, determine the role of adjuvant therapy, and formulate an evidence-informed treatment strategy in the absence of standardized guidelines. This collaborative approach is particularly important for tumors involving the skull base and adjacent critical neurovascular structures, where decisions regarding surgical margins, reconstruction, and adjuvant treatment require careful consideration of both oncologic outcomes and functional preservation.

Complete surgical resection with negative margins is the primary curative strategy. However, because of the proximity to critical neurovascular structures, including the base of the skull, orbits, and cranial nerves, wide local excision is often not feasible. As a result, positive or close margins are common. Despite longstanding misconceptions about sarcoma radioresistance, multiple studies have shown that adjuvant radiation therapy can improve local control, particularly in patients with positive margins [8]. Dose-escalated radiation therapy in the post-operative setting may provide additional benefit in improving locoregional control [9].

Sarcomas are a genetically diverse group of malignancies, often characterized by specific chromosomal translocations, gene rearrangements, or mutations, that aid in classification and may have prognostic or therapeutic relevance. For example, Ewing sarcoma is defined by the EWSR1-FLI1 fusion gene [10], while synovial sarcoma is associated with the SYT-SSX fusion gene [11]. In contrast, LMS demonstrates a complex karyotype with no pathognomonic fusion gene. The most commonly observed genetic alterations include loss-of-function mutations in tumor suppressor genes such as RB1 and PTEN [12]. These changes, along with frequent alterations in TP53 and DNA damage repair pathways, may underlie the poor prognosis of LMS as highlighted in a recent review of immunotherapy trials [13].

LMS of the head and neck is significantly less common than its counterparts arising in the retroperitoneum or uterus. Retroperitoneal LMS frequently originates from large vascular structures such as the inferior vena cava and accounts for up to 69% of retroperitoneal sarcomas [14]. Uterine LMS comprises approximately 7% of soft tissue sarcomas and 1% to 3% of all uterine malignancies [15]. By contrast, LMS of the head and neck is exceedingly rare. In a review by Yadav et al., only 41 cases were identified [16]. A large retrospective review of 352 head and neck sarcomas at the Mayo Clinic identified LMS in only 1.1% of cases [17]. This rarity likely reflects the relative paucity of smooth muscle in head and neck subsites, with oral cavity, paranasal sinuses, and superficial soft tissues (e.g., scalp) being the most commonly involved regions [16].

Diagnosis of LMS in the head and neck is often delayed due to nonspecific clinical presentation and its histologic overlap with other spindle cell tumors. Immunohistochemistry is essential for the accurate diagnosis, with tumor cells typically staining positive for SMA, desmin, and h-caldesmon. The standard of care remains surgical excision with negative margins. Failure of local control is the most common cause of treatment failure, underscoring the importance of achieving adequate surgical margins [6]. Due to the infiltrative nature of LMS and its tendency to extend beyond grossly visible disease, a margin of at least 1 cm is recommended when anatomically feasible [16].

Distant metastases from head and neck LMS are uncommon, with hematogenous spread, most often to the lungs and bones, being the predominant route. Lymph node metastasis (LNM) is rare, with an incidence of 0.9% to 6% across soft tissue sarcomas and only 1.3% in LMS specifically [18]. Notably, prophylactic lymph node dissections have not demonstrated a survival benefit in LMS and are generally not recommended [18].

The role of radiation therapy, both in the adjuvant and neoadjuvant setting, continues to evolve. Preoperative external beam radiation may reduce tumor size, facilitate resection, and minimize the risk of intraoperative tumor seeding [16]. However, the optimal timing, dose, and patient selection criteria remain areas for further study.

## Conclusions

LMS of the SNT is an uncommon and aggressive malignancy, often presenting with non-specific symptoms and locally advanced disease. Surgical resection remains the mainstay of treatment, but negative margins are frequently difficult to achieve due to anatomical constraints. Adjuvant radiation therapy should be considered, particularly in cases of high-risk features such as positive margins. Early diagnosis, multidisciplinary coordination, and individualized treatment planning are critical to improving outcomes in this challenging disease.
